# Synopsis of Human Anatomy

**Published:** 1892-07

**Authors:** 


SYNOPSIS OF HUMAN ANATOMY.—Being a complete compend of anatomy, including the anatomy of the viscera, and numerous tables. By James K. Young, M. D., Instructor in Orthopædic Surgery, and Assistant Demonstrator of Surgery in the University of Pennsylvania; Attending Orthopædic Surgeon, Out-Patients, University Hospital; Fellow of the College of Physicians, etc. A work of 393 pages, illustrated. Published by F. A. Davis, Philadelphia and London.

These two works on anatomy make good companion works for the desk of the student, or those interested in the study of anatomy. Their arrangement and style is different, but both are concise and accurate. The table of contents and general index are particularly well arranged for ready reference. The former covers rather a larger scope than the latter, which is published in the Physician's and Student's Ready Reference Series. Both books are well edited and are fully abreast of the latest teachings in anatomy.

The illustrations are particularly well selected, as we should expect from men who have been teachers long enough to fully know and appreciate the needs of students.

